# Applying the vaginal approach to ovarian cystectomy: current evidence and future applications

**DOI:** 10.2144/fsoa-2020-0082

**Published:** 2020-06-26

**Authors:** Jan F Baekelandt, Jan JA Bosteels

**Affiliations:** 1 Department of Obstetrics & Gynaecology, Imelda Hospital, Bonheiden, Belgium

Applying the vaginal approach to ovarian cystectomy: current evidence and future applications. Galazis *et al.* [1].

4 May 2020

We would like to congratulate Galazis *et al.* on their elegant review paper. The interest in vaginal access surgery other than conventional vaginal hysterectomy is indeed growing rapidly despite the paucity in evidence [2].

Throughout our vaginal natural orifice transluminal endoscopic surgery (vNOTES) research we adhere to the principles of the IDEAL collaboration [3]. The vNOTES hysterectomy results of one single-center, prospective, blinded, randomized controlled trial (HALON trial) [2] have been published and the protocol for a large prospective multicenter randomized controlled trial (RCT) is almost ready. The data for vNOTES adnexectomy of one single-center, prospective, blinded, randomized controlled trial (NOTABLE trial) are being processed and the results are expected to be ready for publication in the second half of 2020 [4]. vNOTES ovarian cystectomy is still in an earlier IDEAL stage so there is, as Galazis *et al.* correctly conclude, currently insufficient evidence supporting the technique. Following the IDEAL principles, the International NOTES Society has maintained a prospective complication database for vNOTES since 2015, where many early adopters of vNOTES enter their data on the various vNOTES procedures, in order to gather evidence and scientifically validate vNOTES.

Based on the current available data on vNOTES, we would like to make two remarks about the paper of Galazis *et al.* [1].

## The authors state

“An increased incidence of bladder injury has been described in vaginal adnexal surgery, particularly in cases of previous cesarean section”

This is incorrect. The referenced paper gives the results of the above mentioned HALON trial comparing vNOTES hysterectomy with total laparoscopic hysterectomy and does not cover vNOTES adnexal surgery. In addition, the HALON trial demonstrated no significant difference in intra-operative complications between both groups and showed a significantly lower rate of postoperative complications in the vNOTES group when compared with the total laparoscopic hysterectomy (TLH) group.

The authors then correctly add: *“however, this is a concern in extensive/advanced NOTES such as hysterectomy”*

We agree that this is only a concern for hysterectomy and not for adnexal surgery. However, we have to await the results of the multicenter prospective complication database or larger studies, before we can scientifically confirm this. In theory, we agree that it is likely that the rate of cystotomy will be higher in vNOTES hysterectomy when compared with vaginal hysterectomy, as more challenging hysterectomies are being addressed via vNOTES (large myomatous uterus, multiple caesarean sections) than is generally the case in total vaginal hysterectomy. These cases involve more challenging anterior colpotomies.

This however does not apply to adnexal surgery as no anterior colpotomy is made in vNOTES adnexal surgery, only a posterior colpotomy and it is extremely unlikely that a surgeon will create a bladder injury in a procedure performed via posterior colpotomy when the uterus sits between the surgical field and the bladder.

## The authors state

“Other approaches, described above, such as laparoscopic-assisted vaginal ovarian cystectomy (VOC), transvaginal-laparoscopic VOC & vaginal NOTES, involve pneumoperitoneum & abdominal incisions, which defeat the purpose of managing benign ovarian cysts vaginally to minimize postoperative pain & improve recovery”

We do not agree with this statement. vNOTES does not involve abdominal incisions.

As for postoperative pain, we agree that further research is needed, but the evidence indicates that it is definitely worth investigating as vNOTES may in fact reduce postoperative pain.

vNOTES does use a pneumoperitoneum and again we need to await the results of the NOTABLE trial to see whether there is a significant difference in postoperative pain and recovery when comparing vNOTES adnexectomy with laparoscopic adnexectomy. If this does show a difference, then prospective RCTs for vNOTES ovarian cystectomy versus laparoscopic ovarian cystectomy still need to be performed. The RCT data on hysterectomy however did show that vNOTES (including pneumoperitoneum and even sham abdominal incisions) significantly reduced postoperative pain, total use of analgesia and facilitated outpatient surgery when compared with total laparoscopic hysterectomy [2].
